# Molecular identification of mosquitoes (Diptera: Culicidae) in southeastern Australia

**DOI:** 10.1002/ece3.2095

**Published:** 2016-03-28

**Authors:** Jana Batovska, Mark J. Blacket, Karen Brown, Stacey E. Lynch

**Affiliations:** ^1^ Department of Economic Development, Jobs, Transport and Resources (DEDJTR)BioSciences ResearchAgriBio Centre for AgriBioscienceBundooraVictoria3083Australia

**Keywords:** Biosecurity, Culicidae, DNA barcoding, mitochondrial COI, species distributions

## Abstract

DNA barcoding is a modern species identification technique that can be used to distinguish morphologically similar species, and is particularly useful when using small amounts of starting material from partial specimens or from immature stages. In order to use DNA barcoding in a surveillance program, a database containing mosquito barcode sequences is required. This study obtained Cytochrome Oxidase I (COI) sequences for 113 morphologically identified specimens, representing 29 species, six tribes and 12 genera; 17 of these species have not been previously barcoded. Three of the 29 species ─ *Culex palpalis*,* Macleaya macmillani,* and an unknown species originally identified as *Tripteroides atripes* ─ were initially misidentified as they are difficult to separate morphologically, highlighting the utility of DNA barcoding. While most species grouped separately (reciprocally monophyletic), the *Cx. pipiens* subgroup could not be genetically separated using COI. The average conspecific and congeneric p‐distance was 0.8% and 7.6%, respectively. In our study, we also demonstrate the utility of DNA barcoding in distinguishing exotics from endemic mosquitoes by identifying a single intercepted *Stegomyia aegypti* egg at an international airport. The use of DNA barcoding dramatically reduced the identification time required compared with rearing specimens through to adults, thereby demonstrating the value of this technique in biosecurity surveillance. The DNA barcodes produced by this study have been uploaded to the ‘Mosquitoes of Australia–Victoria’ project on the Barcode of Life Database (BOLD), which will serve as a resource for the Victorian Arbovirus Disease Control Program and other national and international mosquito surveillance programs.

## Introduction

Vector surveillance requires a rapid and accurate method to identify species of importance. Over 300 species of mosquitoes are known to occur in Australia, many of which have the potential to vector pathogens of disease significant to human and animal health (Ehlersm and Alsemgeest 2011). Surveillance is also conducted for international mosquito vectors, such as *Stegomyia albopicta* (currently exotic) and *St. aegypti* (established in tropical Australia), which pose a considerable public health risk due to the variety of diseases they can transmit. Mosquito identification in Australian surveillance programs currently relies on morphological identification of specimens using dichotomous keys. This traditional approach is time‐consuming, requires specialist knowledge and can be problematic when trying to identify damaged specimens or distinguish morphologically similar species. DNA barcoding is a complementary identification method, which has the potential to overcome these current limitations.

DNA barcoding is a molecular approach to species identification, which involves the use of a short DNA sequence that has much less variance within species than it does between species. To date, molecular studies of endemic Australian mosquitoes have investigated only one genus at a time (Foley et al. 1998, 2007; Hemmerter et al. 2007; Ballard et al. 2009; Puslednik et al. 2012; Endersby et al. 2013; Kassim et al. 2013). These studies have demonstrated the potential of DNA barcoding by further defining geographical distributions and genetic diversity of species. However, they represent only a small minority of the total number of mosquito species in Australia and the data obtained is difficult to compare due to the variety of genetic regions used as DNA barcodes.

**Figure 1:**
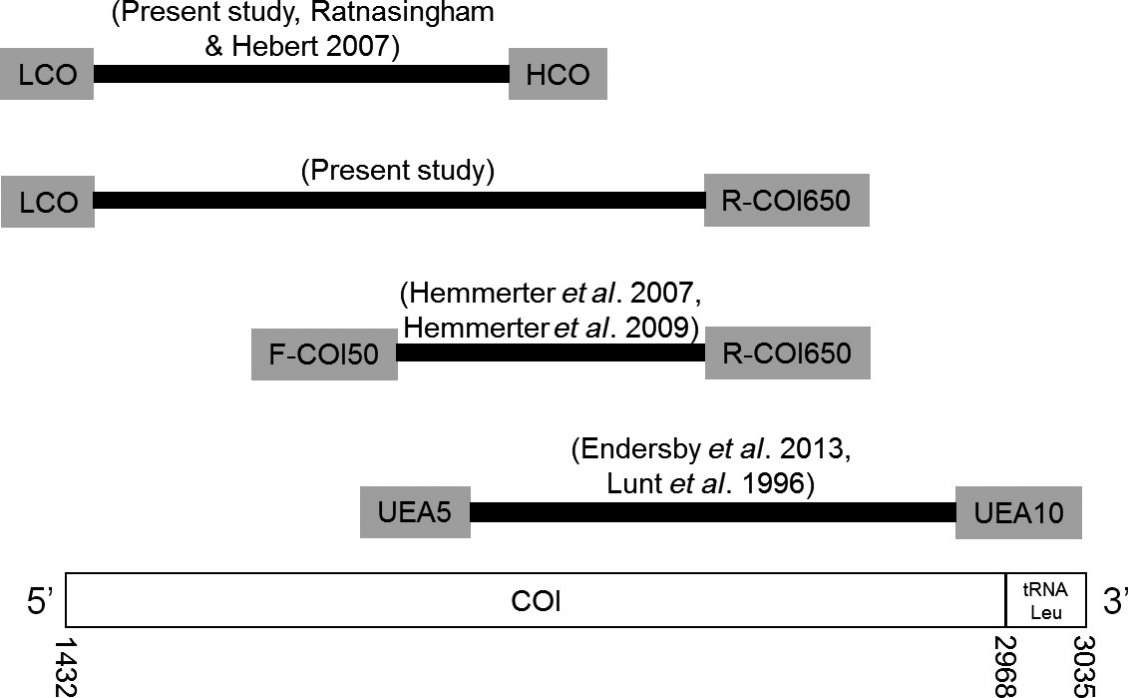
Comparison of primer locations within COI in different mosquito DNA barcoding studies. Mitochondrial gene positions sourced from (Hardy et al. 2014), GenBank accession number: NC_025473.

Currently, the most commonly used barcode region for animals is a 5′‐segment of the mitochondrial gene Cytochrome Oxidase I (COI) called the ‘Universal’ or ‘Folmer’ region. This region is the standard marker chosen by the Barcode of Life Database (BOLD), which is an online platform for collating and curating DNA barcoding information from around the world (Ratnasingham and Hebert 2007). While the majority of mosquito barcoding studies use this region, some studies have used other areas of COI (Fig. 1). Often more than one marker will be used, with both mitochondrial and nuclear genes exhibiting utility in distinguishing species (Lin and Danforth 2004). In mosquito barcoding studies, a variety of nuclear markers have been used, including elongation factor‐1 alpha (EF‐1*α*), acetylcholinesterase 2 (ace‐2), alpha amylase, zinc finger, and internal transcribed spacer subunit 2 (ITS2) (Foley et al. 2007; Hasan et al. 2009; Hemmerter et al. 2009; Puslednik et al. 2012). Using multiple genes can help to distinguish members of species complexes and subgroups, which are closely related species that may not be genetically distinct when using just one barcoding region (Foster et al. 2013; Jiang et al. 2014).

In order to efficiently use DNA barcoding as a mosquito identification method within a surveillance program, a barcode library based on accurate identifications must first be established. For example, 36 different endemic mosquito species have been previously detected in a mosquito surveillance program in Victoria, Australia (Lynch et al. 2015 unpublished data), however only 10 of these species have COI sequences publically available in GenBank and BOLD, and only eight species (<25%) are sampled from Australia (accessed 05.11.15). The creation of a barcode library of the mosquitoes commonly collected in temperate southeastern Australia will allow DNA barcoding to become a useful identification technique, forming the foundation for a larger library of mosquito sequences from all around Australia, as well as contributing to reference mosquito sequences available internationally. The development of a regionally targeted barcode library will help improve the accuracy of mosquito identification as currently DNA barcoding is an under‐utilized technique in mosquito vector surveillance programs.

Barcoding a broad range of mosquito species also allows insight into the composition of genera. In recent years, mosquito taxonomy has undergone a series of debated reclassifications, with the tribe Aedini undergoing the most changes (Reinert and Harbach 2005; Reinert et al. 2009). Traditional classification of mosquitoes is based primarily on similarities in morphology, resulting in broad genus groups which under‐represent the true diversity, with more recent phylogenetic studies suggesting many of these genera contain paraphyletic and polyphyletic taxa (Harbach 2007). Genetic techniques are considered to be relatively free from the subjectivity of identifying morphological features and can reveal the presence of cryptic species complexes that are often overlooked (e.g., Hemmerter et al. 2007). As such, barcoding as a method for identifying mosquitoes is vital to the accuracy of a surveillance program.

In this study, we primarily sought to improve current vector surveillance programs by expanding the COI DNA barcode information available for endemic mosquito species in Australia by generating a barcode library for 26 species collected from temperate southeastern Australia. The DNA barcode library was supported by diagnostic specimen images and collection details uploaded to BOLD as part of the “Mosquitoes of Australia–Victoria” (MOAV) project, adding temperate southeastern Australia to the Mosquitoes of the World campaign. We also aimed to test the utility of DNA barcoding in biosecurity scenarios by identifying a mosquito egg intercepted at an airport. Lastly, we evaluated the use of a larger COI fragment as a barcode to overlap with data from previous studies that have examined various regions of COI (Fig. 1), and discuss the relationships between different mosquito species and the composition of mosquito genera.

## Materials and Methods

### Specimen collection and identification

**Figure 2:**
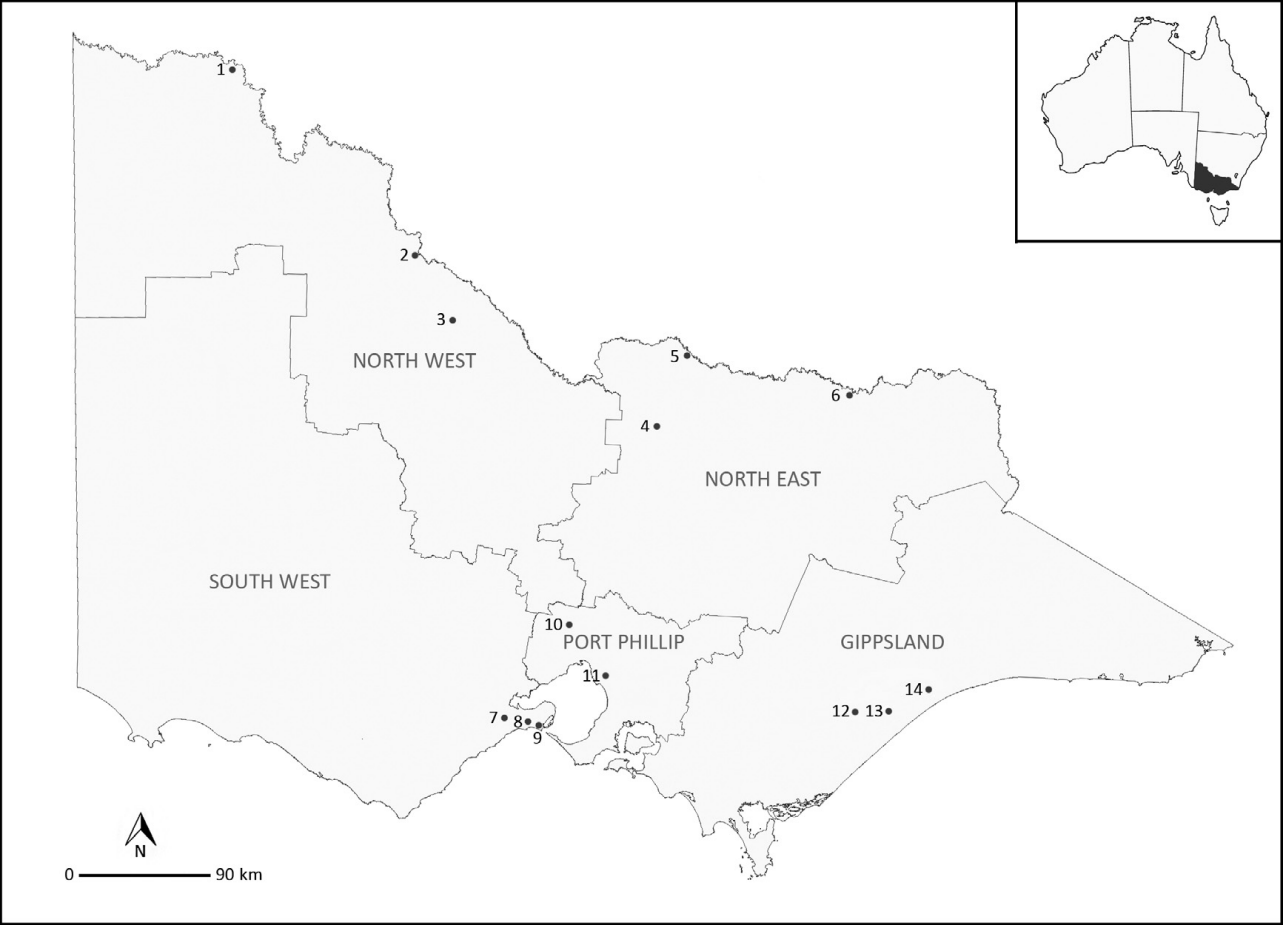
Map of mosquito trapping locations around Victoria State, Australia. 1 =  Mildura, 2 =  Swan Hill, 3 =  Kerang, 4 =  Toolamba, 5 =  Cobram, 6 =  Wodonga, 7 =  Armstrong Creek, 8 =  Ocean Grove, 9 =  Point Lonsdale, 10 =  Melbourne Airport, 11 =  Heatherton, 12 =  Clydebank, 13 =  Meerlieu, 14 =  Paynesville.

Adult mosquitoes were collected using a combination of CO_2_‐baited encephalitis virus surveillance traps (Australian Entomological Supplies, Murwillumbah, Australia) and Biogents Sentinel traps (Biogents, Regensburg, Germany) as part of the Victorian Arbovirus Disease Control Program. The traps were located in 13 different sites within five regions around Victoria (Fig. 2). The majority of specimens were trapped during the 2013/2014 season and stored dry at −20°C prior to analysis. For uncommon mosquito species, pinned specimens stored dry in the Victorian Agricultural Insect Collection (VAIC) were also used for the study (Table 1). In addition to the adult mosquitoes, one *St. aegypti* egg discovered by the Australian Department of Agriculture at Melbourne Airport in January 2015 was used in this study (stored in 95% ethanol at room temperature).

**Table 1:** Details of mosquito specimens used in the study

ID	n =	Species name	Traditional name	City/town	Latitude	Longitude	Accession number
4333	1	*Aedeomyia venustipes*	*Aedeomyia venustipes*	Paynesville	37 53 59.83 S	147 43 08 E	KU494978
4560	2	*Aedeomyia venustipes*	*Aedeomyia venustipes*	Meerlieu	37 59 58.64 S	147 22 21 E	KU494977, KU494979
4309	3	*Anopheles annulipes*	*Anopheles annulipes*	Wodonga	36 06 22.60 S	146 52 26 E	KU494980–83
4311	2	*Anopheles annulipes*	*Anopheles annulipes*	Clydebank	38 04 38.7 S	147 14 40 E	KU494981, KU494984
4315	2	*Coquillettidia linealis*	*Coquillettidia linealis*	Paynesville	37 53 59.83 S	147 43 08 E	KU494987–88
4332	1	*Coquillettidia linealis*	*Coquillettidia linealis*	Paynesville	37 53 59.83 S	147 43 08 E	KU494985
4340	1	*Coquillettidia linealis*	*Coquillettidia linealis*	Kerang	35 43 00.71 S	143 54 38 E	KU494986
4314^b^	1	*Culex annulirostris*	*Culex annulirostris*	Wodonga	36 05 21.24 S	146 49 07 E	KU494989
4322^c^	1	*Culex annulirostris*	*Culex annulirostris*	Toolamba	36 30 43.6 S	145 18 30 E	KU494993
4324^b^	1	*Culex annulirostris*	*Culex annulirostris*	Swan Hill	35 18 34.17 S	143 34 01 E	KU494994
4342^b^	1	*Culex annulirostris*	*Culex annulirostris*	Cobram	35 54 16.12 S	145 38 30 E	KU494995
4343^c^	1	*Culex annulirostris*	*Culex annulirostris*	Mildura	34 09 42.56 S	142 09 30 E	KU494990
4344^b^	1	*Culex annulirostris*	*Culex annulirostris*	Toolamba	36 30 43.6 S	145 18 30 E	KU494991
4345^b^	1	*Culex annulirostris*	*Culex annulirostris*	Kerang	35 43 00.71 S	143 54 38 E	KU494992
4310	2	*Culex australicus*	*Culex australicus*	Toolamba	36 30 43.6 S	145 18 30 E	KU494999, KU495000
4326	3	*Culex australicus*	*Culex australicus*	Meerlieu	38 01 15.49 S	147 16 31 E	KU494996–98
4307	4	*Culex globocoxitus*	*Culex globocoxitus*	Meerlieu	38 01 15.49 S	147 16 31 E	KU495001–04
4312	2	*Culex pipiens* form *molestus*	*Culex pipiens* form *molestus*	Clydebank	38 04 38.7 S	147 14 40 E	KU495005–06
4339	2	*Culex pipiens* form *molestus*	*Culex pipiens* form *molestus*	Kerang	35 44 25.16 S	143 56 28 E	KU495007–08
4348	5	*Culex cylindricus*	*Culex cylindricus*	Wodonga	36 05 21.24 S	146 49 07 E	KU495009–13
4682^d^	2	*Culex palpalis*	*Culex palpalis*	Paynesville	37 53 59.83 S	147 43 08 E	KU495015–16
4683^d^	1	*Culex palpalis*	*Culex palpalis*	Kerang	35 42 25.66 S	143 54 24 E	KU495014
4684^d^	1	*Culex palpalis*	*Culex palpalis*	Mildura	34 14 58.62 S	142 13 06 E	KU495017
4318	5	*Culex quinquefasciatus*	*Culex quinquefasciatus*	Cobram	35 54 16.12 S	145 38 30 E	KU495018–22
4903^a^	1	*Culiseta inconspicua*	*Culiseta inconspicua*	Meerlieu	38 01 15.49 S	147 16 31 E	KU495024
4904^a^	1	*Culiseta inconspicua*	*Culiseta inconspicua*	Meerlieu	38 01 15.49 S	147 16 31 E	KU495023
4329	1	*Dobrotworskyius alboannulatus*	*Aedes alboannulatus*	Armstrong Creek	38 14 09.86 S	144 22 14 E	KU495027
4331	3	*Dobrotworskyius alboannulatus*	*Aedes alboannulatus*	Ocean Grove	38 15 00.77 S	144 31 32 E	KU495025–26, KU495028
4327	2	*Dobrotworskyius rubrithorax*	*Aedes rubrithorax*	Toolamba	36 30 43.6 S	145 18 30 E	KU495031–32
4681	1	*Dobrotworskyius rubrithorax*	*Aedes rubrithorax*	Armstrong Creek	38 14 09.86 S	144 22 14 E	KU495029
4902^a^	1	*Dobrotworskyius rubrithorax*	*Aedes rubrithorax*	Toolamba	36 30 43.6 S	145 18 30 E	KU495030
4551	1	*Macleaya macmillani*	*Aedes macmillani*	Meerlieu	38 03 39.42 S	147 25 39 E	KU495033
4334	1	*Macleaya tremula*	*Aedes tremulus*	Mildura	34 09 42.56 S	142 09 30 E	KU495036
4556	1	*Macleaya tremula*	*Aedes tremulus*	Mildura	34 10 43.34 S	142 08 12 E	KU495034
4669^a^	1	*Macleaya tremula*	*Aedes tremulus*	Mildura	34 12 22.88 S	142 08 03 E	KU495035
4905^a^	1	*Macleaya tremula*	*Aedes tremulus*	Mildura	34 09 42.56 S	142 09 30 E	KU495037
4335	1	*Macleaya wattensis*	*Aedes wattensis*	Swan Hill	35 18 34.17 S	143 34 01 E	KU495039
4558	1	*Macleaya wattensis*	*Aedes wattensis*	Cobram	35 52 30.9 S	145 34 09 E	KU495038
4668^a^	1	*Macleaya wattensis*	*Aedes wattensis*	Cobram	35 53 56.64 S	145 37 12 E	KU495040
4323	1	*Mucidus alternans*	*Aedes alternans*	Toolamba	36 30 43.6 S	145 18 30 E	KU495045
4328	2	*Mucidus alternans*	*Aedes alternans*	Swan Hill	35 18 34.17 S	143 34 01 E	KU495041, KU495044
4336	2	*Mucidus alternans*	*Aedes alternans*	Swan Hill	35 18 34.17 S	143 34 01 E	KU495042–43
4308	5	*Ochlerotatus bancroftianus*	*Aedes bancroftianus*	Wodonga	36 06 22.60 S	146 52 26 E	KU495046–50
4304	2	*Ochlerotatus camptorhynchus*	*Aedes camptorhynchus*	Meerlieu	38 01 15.49 S	147 16 31 E	KU495054–55
4330	1	*Ochlerotatus camptorhynchus*	*Aedes camptorhynchus*	Point Lonsdale	38 17 12.4 S	144 36 26 E	KU495053
4341	1	*Ochlerotatus camptorhynchus*	*Aedes camptorhynchus*	Mildura	34 10 43.34 S	142 08 12 E	KU495052
4347	1	*Ochlerotatus camptorhynchus*	*Aedes camptorhynchus*	Mildura	34 09 42.56 S	142 09 30 E	KU495051
4303	1	*Ochlerotatus mallochi*	*Aedes mallochi*	Mildura	34 14 58.62 S	142 13 06 E	KU495058
4325	1	*Ochlerotatus mallochi*	*Aedes mallochi*	Mildura	34 14 58.62 S	142 13 06 E	KU495059
4553	1	*Ochlerotatus mallochi*	*Aedes mallochi*	Mildura	34 11 59.79 S	142 12 17 E	KU495057
4554	1	*Ochlerotatus mallochi*	*Aedes mallochi*	Mildura	34 11 59.79 S	142 12 17 E	KU495056
4555	1	*Ochlerotatus mallochi*	*Aedes mallochi*	Mildura	34 09 42.56 S	142 09 30 E	KU495060
4305	5	*Ochlerotatus sagax*	*Aedes sagax*	Kerang	35 42 25.66 S	143 54 24 E	KU495061–65
4302	5	*Ochlerotatus theobaldi*	*Aedes theobaldi*	Toolamba	36 30 43.6 S	145 18 30 E	KU495066–70
4301	5	*Ochlerotatus vittiger*	*Aedes vittiger*	Swan Hill	35 18 34.17 S	143 34 01 E	KU495071–75
4319	3	*Rampamyia notoscripta*	*Aedes notoscriptus*	Cobram	35 52 30.9 S	145 34 09 E	KU495076, KU495079–80
4337	2	*Rampamyia notoscripta*	*Aedes notoscriptus*	Paynesville	37 53 59.83 S	147 43 08 E	KU495077–78
4689	1	*Stegomyia aegypti*	*Aedes aegypti*	Melbourne	37 40 07.69 S	144 50 27 E	KU495081
4665^a^	1	*Stegomyia albopicta*	*Aedes albopictus*	Heatherton	37 56 33.59 S	145 05 35 E	KU495082
4313	1	*Tripteroides atripes*	*Tripteroides atripes*	Mildura	34 09 42.56 S	142 09 30 E	KU495088
4316	3	*Tripteroides* sp.	*Tripteroides* sp.	Paynesville	37 53 59.83 S	147 43 08 E	KU495083–84, KU495087
4320	1	*Tripteroides atripes*	*Tripteroides atripes*	Mildura	34 14 58.62 S	142 13 06 E	KU495085
4661^a^	1	*Tripteroides atripes*	*Tripteroides atripes*	Toolamba	34 09 42.56 S	142 09 30 E	KU495086
4349	1	*Tripteroides tasmaniensis*	*Tripteroides tasmaniensis*	Paynesville	37 53 59.83 S	147 43 08 E	KU495089

a Dry‐pinned specimens stored in the VAIC.

b Ann‐S‐AUS lineage.

c Ann‐AUS lineage.

d Pal‐S‐AUS lineage (in relation to findings from Hemmerter et al. 2007).

Mosquitoes were morphologically identified using taxonomic keys (Dobrotworsky 1965; Russell 1996). Composite auto‐montage images were taken of a representative specimen from each species using a Leica M205 C microscope and camera, and the images were submitted to BOLD as part of the Mosquitoes of Australia–Victoria (MOAV) project. The representative specimens were then pinned as voucher specimens and stored in the VAIC. Database numbers for all examined specimens are included in MOAV and listed in Table 1.

In total, 113 mosquito specimens were used, comprising of 12 genera and 29 species (Table 1). In light of the results found in this study, the currently accepted generic designations from the Mosquito Taxonomic Inventory's Valid Species List (http://mosquito-taxonomic-inventory.info/valid-species-list, accessed 15 September 2015) and the Atlas of Living Australia (http://www.ala.org.au/, accessed 15 September 2015) have been used instead of traditional species names, however both are provided in Table 1 to avoid confusion and for comparison with previous literature. The Atlas of Living Australia lists *Culex molestus* as a valid species due to its widespread usage in Australia (Russell 2012), however *Cx. pipiens* form *molestus* is used in this paper as *Cx. molestus* is a physiological variant of *Cx. pipiens* (Harbach et al. 1984).

### DNA isolation

A leg was removed from each frozen mosquito and half of the dry‐pinned specimens for DNA isolation. Each leg was homogenized using beads in 20 *μ*L of proteinase K, then incubated in 50 *μ*L of Buffer ATL (QIAGEN, Hilden, Germany) for 60 min at 56°C. Of this lysate, 50 *μ*L was used for total DNA extraction in a MagMAX Express Magnetic Particles Processor using the MagMAX DNA Multi‐Sample Kit (Life Technologies, Gaithersburg, MD, USA). The extraction procedure followed the manufacturer's instructions with the exception that RNase A mix was not used. Approximately 50 *μ*L of total DNA was extracted for each sample. All DNA isolates were stored at −20°C.

An alternative DNA extraction method was employed for the *St. aegypti* egg and the other half of the dry‐pinned specimens. The egg was taken out of the 95% ethanol, and a leg was removed from each pinned specimen. The egg and legs were homogenized individually in 20 *μ*L of proteinase K, then incubated in 180 *μ*L of Buffer ATL for 60 min at 56°C. DNA was extracted using a DNeasy Blood & Tissue Kit (QIAGEN) according to the manufacturer's instructions, including a double final elution step. For each sample, a total of 100 *μ*L of DNA was extracted. All DNA isolates were stored at −20°C.

### COI amplification, sequencing, and data analysis

An 840 bp COI fragment was amplified using the primer pairs LCO1490 (Folmer et al. 1994) and R‐COI650 (Hemmerter et al. 2007). Two dry‐pinned specimens appeared to have degraded DNA due to prolonged storage, hence a smaller 648 bp COI fragment was amplified using the ‘Universal’ primer pair LCO1490 and HCO2198 (Folmer et al. 1994). The total PCR volume was 25 *μ*L and consisted of 15.3 *μ*L 1 × bovine serum albumin (BSA), 2.5 *μ*L 10 × ThermoPol Reaction Buffer (New England Biolabs, Beverly, MA, USA), 2 *μ*L 2.5 *μ*M dNTPs, 1.25 *μ*L of each 10 *μ*M/L primer, 0.2 *μ*L 1.0 U *Taq* DNA Polymerase, and 2.5 *μ*L template DNA. Unsuccessful PCRs were repeated using 5 *μ*L of template DNA and a proportionally adjusted BSA volume. The COI PCR cycle was as follows: 94°C for 2 min, 40 cycles of 94°C for 30 s, 49°C for 45 s and 72°C for 45 s, then finally 72°C for 1 min. The PCR products were verified on a 2% agarose gel.

Size‐verified COI PCR products were enzymatically purified and sequenced commercially in both directions on an ABI3730XL by Macrogen Inc. (Korea). Forward and reverse sequences were assembled and edited in Geneious version 8.1 (http://www.geneious.com, Kearse et al. 2012). In a small number of specimens (see Results), clear double bases were called by eye using International Union of Pure Applied Chemistry (IUPAC) ambiguity codes. Edited sequences (744 bp) were aligned with ClustalW, sequence divergence was calculated (p‐distance values), and a bootstrap neighbor‐joining tree (1000 replicates) was created using MEGA version 6 (Tamura et al. 2007). All COI sequences have been uploaded to the MOAV project in BOLD and deposited in GenBank; accession numbers are provided in Table 1 and in the Data Accessibility section.

## Results

### Comparison between DNA barcodes and morphological assessments

Using taxonomic keys, 26 species were morphologically identified from the 113 mosquito specimens. Molecular identification revealed a further three species, resulting in a total of 29 species. The three additional species were *Cx. palpalis* (originally identified as *Cx. annulirostris*), *Macleaya macmillani* (originally identified as *Mc. tremula*), and an unknown species (originally identified as *Tripteroides atripes*, referred to here as *Tp*. sp.).

### COI analyses

All 113 mosquito specimens had the COI target gene sequenced and were used in the final analysis. The sequences were AT‐rich, with an average of 69.6% AT content for all codons. *Coquillettidia linealis* had the lowest AT content with 65.9%, whereas *Cx. cylindricus* had the most with 71.2%. None of the sequences contained indels or stop codons. *Culiseta inconspicua* was the only species with COI sequences containing ambiguous bases, with 1.3–2.6% of the final 744 bases being called as heterozygous double bases, suggesting the likely presence of pseudogenes (i.e., numts) in this species.

**Figure 3:**
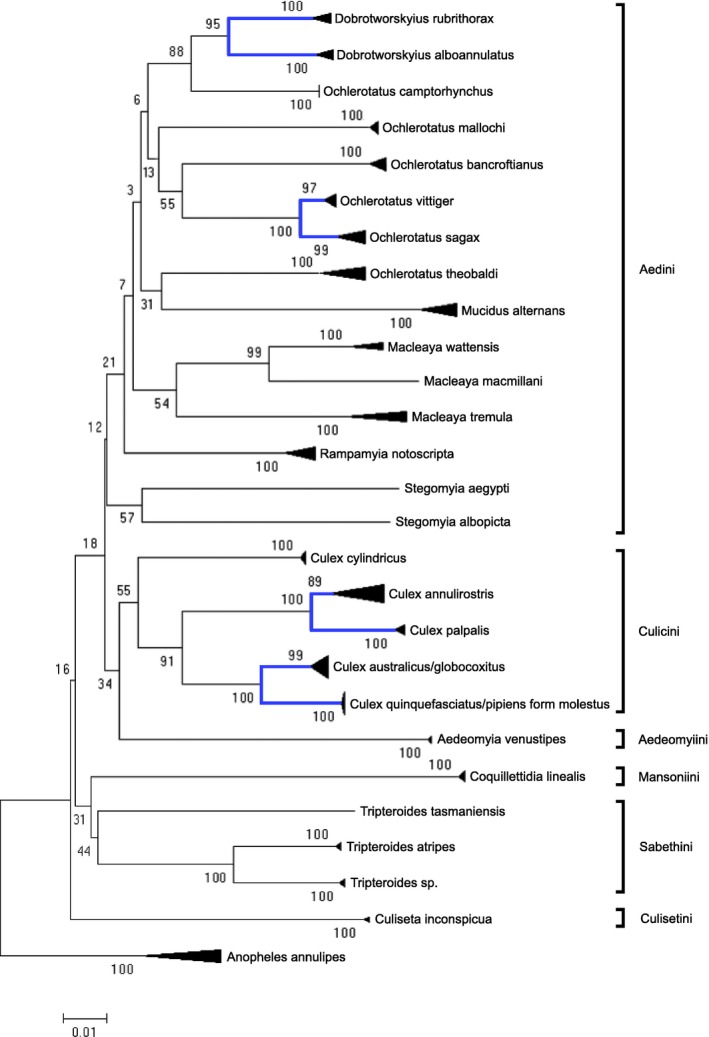
A summarized neighbor‐joining tree, with bootstrap support values (%), based on p‐distance comparisons between COI sequences from 113 mosquito specimens. The groups highlighted in blue are species complexes or subgroups (defined as species with ≤4% divergence between them). The brackets indicate the tribal groups.

**Figure 4:**
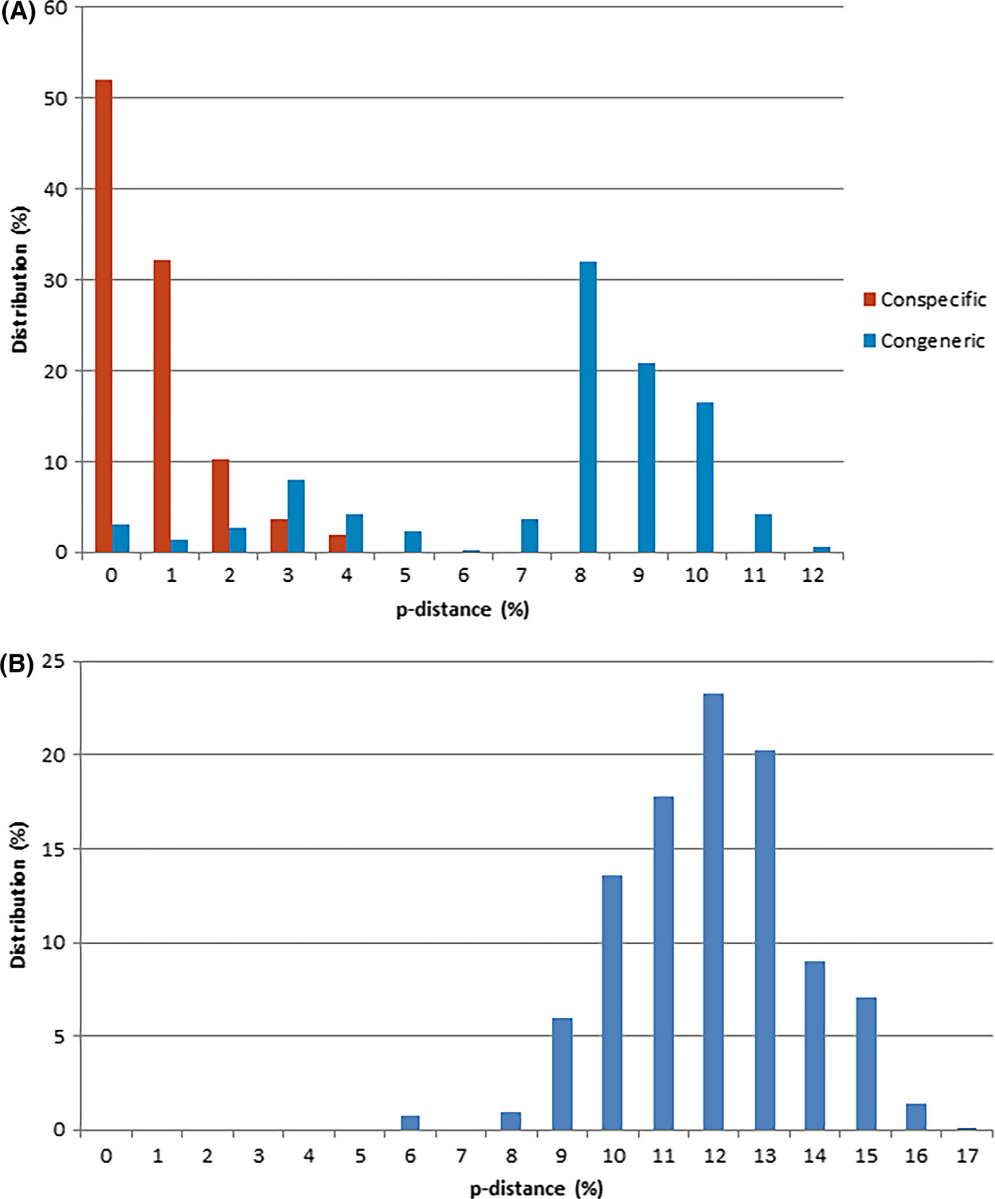
Distribution of percentage difference (p‐distances) for COI in different taxonomic categories. (A) Comparison of conspecific and congeneric differences in 113 COI samples. (B) Differences in 113 COI samples between genera.

The neighbor‐joining analyses of the COI sequences revealed that all 29 species grouped separately, except for *Cx. quinquefasciatus* and *Cx. pipiens* form *molestus*, and *Cx. australicus* and *Cx. globocoxitus* (Fig. 3). These four species are part of the *Cx. pipiens* subgroup, whereas *Cx. quinquefasciatus*,* Cx. pipiens* form *molestus* and *Cx. australicus* form the *Cx. pipiens* complex (Smith and Fonseca 2004). The average conspecific p‐distance was 0.8% (range 0–4.2%), compared to 7.6% (range 0–12.3%) for congeneric divergence (Fig. 4A). Species with less than 4% divergence were considered to be species complexes. Between genera, p‐distances increased to an average of 12% (range 5.5–16.9%) (Fig. 4B).

The egg intercepted at the Melbourne Airport was identified through DNA sequence analysis as *St. aegypti*. The COI sequence had 100% similarity to other *St. aegypti* COI sequences stored on BOLD.

## Discussion

The primary aim of this study was to create a DNA barcode library for the common mosquito species found in temperate south eastern Australia. The majority of the 26 barcoded species formed distinctive clusters, confirming the utility of the DNA barcoding method in mosquito surveillance programs. Furthermore, the sequencing results revealed additional species that had been initially morphologically misidentified.

### Cryptic species

This study is the first to report the detection of *Cx. palpalis* (originally identified as *Cx. annulirostris*) in Victoria. *Culex palpalis* was detected from trapping locations in the North West and Gippsland regions of the state (Table 1, Fig. 2), extending the geographical distribution of this species along the entire Australian East Coast (Hemmerter et al. 2007). Morphological features were consistent with those described by Jansen et al. (2013). Another species that was discovered after molecular identification was *Mc. macmillani* (originally identified as *Mc. tremula*). The trapping location in the Gippsland region corresponds with the distribution described by (Dobrotworsky (1965). The final additional species was originally identified as *Tp. atripes*, however it grouped separately (>5% divergence, Fig. 3) and showed a distinct geographical distribution (*Tp. atripes* specimens were collected in the inland northern Victoria region, whereas the undetermined *Tp*. specimens were from the coastal Gippsland region) suggesting the presence of a cryptic species. The two groups could not be separated morphologically due to damaged specimens, however *Tp. marksae* is morphologically similar to *Tp. atripes* and is only known from the Gippsland region (Dobrotworsky 1965), so might be a possible candidate for the undetermined *Tp*. species. Additional sampling should help to clarify species identifications by providing specimens in better morphological condition.

### Species complexes and subgroups

Whereas the majority of species clustered separately, COI was not able to distinguish members of the *Cx. pipiens* subgroup (Fig. 3), which consists of morphologically distinct species. *Culex globocoxitus* and *Cx. australicus* had 0–1% divergence, while *Cx. quinquefasciatus* and *Cx. pipiens* form *molestus* had 0%. The genetic similarity within the *Cx. pipiens* subgroup is well documented, and various molecular techniques have been developed to distinguish between these species, including the use of the ace‐2 gene (Smith and Fonseca 2004; Lee et al. 2012; Al‐Hussaini et al. 2013; Laurito et al. 2013). *Culex australicus* is considered to be part of the *Cx. pipiens* complex (Smith and Fonseca 2004), however its similarity to *Cx. globocoxitus* suggests it may only belong to the subgroup.

Other species groups with low congeneric divergence included *Ochlerotatus sagax* and *Oc. vittiger* (2–3%), *Cx. annulirostris* and *Cx. palpalis* (3–4%), and *Dobrotworskyius alboannulatus* and *Db. rubrithorax* (4–5%) (Fig. 3). Along with the *Cx. pipiens* subgroup, these groups account for all of the overlap between conspecific and congeneric differences seen in Figure 4A. Species complexes are known to create issues with applying the ‘barcoding gap’ and the ability to separate species (Čandek and Kuntner 2015). However, despite the low divergence between these groups, all species other than those in the *Cx. pipiens* subgroup clustered separately, thereby confirming the diagnostic capability of DNA barcoding using COI.

### Generic designations

Although phylogenetic analyses were not performed in the current study, the clustering of the genera found here appears to mostly agree with the reclassification in the tribe Aedini made by Reinert et al. (2009). The genera *Dobrotworskyius*,* Mucidus*,* Macleaya*,* Rampamyia,* and *Stegomyia* all appear monophyletic and distinct from one another, whereas *Ochlerotatus* was not recovered as a single group, with *Oc. camptorhynchus* and *Oc. theobaldi* distinct from other species. However, it is difficult to make conclusions from this study about what the relationships between species means for mosquito taxonomy, as only one marker and a select few species from each genus were used. Far broader and thorough sampling using multiple markers, if not entire genome sequences, is required to make definitive conclusions about mosquito taxonomy (Foster et al. 2013; Wilkerson et al. 2015).

### Utility of DNA barcoding in mosquito surveillance programs

The barcoding of 29 mosquito species from temperate southeastern Australia has expanded the database of barcodes available on the Mosquitoes of the World campaign (BOLD). Targeted barcode libraries allow DNA barcoding to become a useful tool for vector surveillance programs, and countries such as Belgium, China, Canada, India, and Singapore have established barcode libraries for their regions (Cywinska et al. 2006; Kumar et al. 2007; Wang et al. 2012; Chan et al. 2014; Versteirt et al. 2015). These studies have all utilized the ‘Universal’ COI region as a DNA barcode, making their data compatible with the data from this study.

The use of a larger COI fragment allows our data to also be compatible with other studies (Fig. 1). The successful amplification of the larger fragment in 111 of the 113 specimens suggests the primer pair used in this study is suitable for DNA barcoding. For comparable data, it is recommended the larger COI fragment be used in studies investigating species that have had the central region of the COI gene previously sequenced, such as *Cx. annulirostris* (Hemmerter et al. 2007). However, studies using specimens with potentially degraded DNA, such as dry‐pinned reference specimens, should use the ‘Universal’ region as it has higher amplification success and is the region used by the majority of DNA barcoding studies worldwide (Ratnasingham and Hebert 2007).

Vector surveillance is often conducted at high‐risk international ports worldwide due to the increasing threat of invasive exotic mosquitoes such as *St. aegypti* and *St. albopicta*. These mosquitoes transmit nonendemic agents of diseases such as dengue, chikungunya, and yellow fever and pose a significant public health risk (Richards et al. 2012). This study has demonstrated an additional biosecurity application of DNA barcoding with the screening of a single egg to confirm the presence of *St. aegypti* at an international port in Melbourne. Unlike morphological identification, DNA barcoding does not require the egg to be hatched, thereby reducing the interception response times and helping to prevent the establishment of exotic mosquitoes.

## Conclusions

In summary, this study established the utility of DNA barcoding in vector surveillance through generating a regionally targeted barcode library for mosquitoes found in temperate southeastern Australia. This barcode library will enable the use of DNA barcoding as an additional identification tool in vector surveillance programs and can continue to be built upon within Australia and internationally. The ability to identify species from any life stage, including eggs, means DNA barcoding is not only useful in surveillance programs, but also biosecurity operations. Future applications of this approach should involve barcoding more species and adding other genetic markers that increase the discriminatory power of this identification method. DNA barcoding could also be utilized with next‐generation sequencing to identify large numbers of mosquitoes at one time (i.e., bulk samples), thereby significantly lowering the processing time involved in species identification (McCormack et al. 2013). The accuracy and versatility of DNA barcoding as a species identification tool makes it an essential part of vector surveillance and will continue to grow in value as further barcode libraries and resources are developed.

## Data Accessibility

DNA sequences are stored on GenBank (accessions KU494977–KU495089). Specimen information, images and DNA sequences were uploaded to BOLD as part of the Mosquitoes of Australia–Victoria project (accessions MOAV001‐15–MOAV116‐15). The phylogenetic data are available on TreeBASE (accession S18559).
